# CD147 Is Essential for the Development of Psoriasis via the Induction of Th17 Cell Differentiation

**DOI:** 10.3390/ijms23010177

**Published:** 2021-12-24

**Authors:** Aoi Okubo, Youhei Uchida, Yuko Higashi, Takuya Sato, Youichi Ogawa, Akihiro Ryuge, Kenji Kadomatsu, Takuro Kanekura

**Affiliations:** 1 Department of Dermatology, Kagoshima University Graduate School of Medical and Dental Sciences, Kagoshima 890-0075, Japan; uchi-y@m2.kufm.kagoshima-u.ac.jp (Y.U.); higashiy@m.kufm.kagoshima-u.ac.jp (Y.H.); takurok@m2.kufm.kagoshima-u.ac.jp (T.K.); 2 Department of Dermatology, Faculty of Medicine, University of Yamanashi, Yamanashi 409-3898, Japan; d17sm039@yamanashi.ac.jp (T.S.); yogawa@yamanashi.ac.jp (Y.O.); 3 Department of Nephrology, Nagoya University Graduate School of Medicine, Aichi 466-8560, Japan; ryuge0120@yahoo.co.jp; 4 Department of Biochemistry, Nagoya University Graduate School of Medicine, Aichi 466-8560, Japan; kkadoma@med.nagoya-u.ac.jp

**Keywords:** CD147/Basigin, psoriasis, Th17, monocarboxylate transporter (MCT), glycolysis

## Abstract

Th17 cells play an important role in psoriasis. The differentiation of naïve CD4^+^ T cells into Th17 cells depends on glycolysis as the energy source. CD147/basigin, an integral transmembrane protein belonging to the immunoglobulin superfamily, regulates glycolysis in association with monocarboxylate transporters (MCTs)-1 and -4 in cancer cells and T cells. We examined whether CD147/basigin is involved in the pathogenesis of psoriasis in humans and psoriasis-model mice. The serum level of CD147 was increased in patients with psoriasis, and the expression of CD147 and MCT-1 was elevated in their dermal CD4^+^ RORγt^+^ T cells. In vitro, the potential of naïve CD4^+^ T cells to differentiate into Th17 cells was abrogated in CD147^−/−^ T cells. Imiquimod (IMQ)-induced psoriatic dermatitis was significantly milder in CD147^−/−^ mice and bone marrow chimeric mice lacking CD147 in the hematopoietic cells of myeloid lineage. These findings demonstrate that CD147 is essential for the development of psoriasis via the induction of Th17 cell differentiation.

## 1. Introduction

Psoriasis, a chronic inflammatory keratotic dermatosis, is characterized by recurrent episodes of sharply demarcated scaly erythematous plaques. The molecular and cellular mechanisms underlying the immunopathogenesis of psoriasis are understood, and recent evidence indicates that Th17 cells and the related signaling pathways play pivotal roles in the development of psoriasis [1,2]. Interleukin (IL)-17, the major product of Th17 cells, stimulates keratinocytes to produce chemokines, cytokines, and proinflammatory mediators such as IL-6, IL-8, GM-CSF, CXCL10, and CCL20, which are responsible for the development of psoriasis [3]. Th17 cells are a distinct T cell subset derived from naïve CD4^+^ helper T cells; the mechanism underlying Th17 cell differentiation is known. The combination of IL-6 and transforming growth factor-β (TGF-β) activates the retinoic acid receptor-related orphan nuclear receptor γt (RORγt), which is the key transcription factor directing the differentiation of Th17 cells [4].

The energy for the differentiation of effector T cells is obtained from glycolysis [5]. The detailed metabolic system of glycolysis in cancer cells has been elucidated. Under physiological conditions, cellular energy is obtained through mitochondrial oxidative phosphorylation, while glycolysis involves anaerobic enzymatic conversions. However, cancer cells depend on glycolysis, even under aerobic conditions, because of the rapid energy production. The cancer cells take up excess glucose, which is enzymatically converted to pyruvate and adenosine triphosphate (ATP) to fuel the pathophysiological activities of cellular growth and proliferation [6]. In aerobic glycolysis in cancer cells, pyruvate is converted to lactic acid, which is excreted from the cytoplasm into the surrounding extracellular microenvironment through the proton-linked/lactate co-transporters, monocarboxylate transporters (MCTs). MCTs form a solute carrier family comprising of 14 members, MCT-1 to MCT-I4. They facilitate proton-linked monocarboxylate transport and play a critical role in glycolysis, by regulating lactic acid transport.

CD147/basigin (CD147) is an integral plasma membrane protein belonging to the immunoglobulin superfamily [7,8]. CD147 is ubiquitously expressed in various cells, including epidermal keratinocytes and lymphocytes. In the K5-promoter transgenic mice designed to overexpress CD147 in epidermal keratinocytes, epidermal CD147 plays a role in the pathogenesis of psoriasis via the production of IL-22 and subsequent Stat3 activation [9].

CD147 exerts pleiotropic functions by binding to various molecules, including MCTs [10]. It associates with two members of the MCT family, MCT-1 and MCT-4, and chaperones their correct localization on the plasma membrane and their catalytic activity [11]. We demonstrated that CD147 colocalizes with MCT-1 and MCT-4 on the plasma membrane and regulates glycolysis in human malignant melanoma (MM) cells [12]. In various cancers, including breast and colon cancers, CD147 plays a role in glycolysis [13,14]. In addition, Le Floch et al. demonstrated that the protumoral action of CD147 is to control the energetics of glycolytic tumors via MCT1/MCT4 activity [15].

Aerobic glycolysis is also important for the differentiation and function of T cells [5]. The differentiation of CD4^+^ T cells into distinct subsets requires aerobic glycolysis; Th1, Th2, and Th17 cells are highly glycolytic [16]. Lactic acid efflux from T cells is mediated by MCT-1. In addition, MCT-1 influences T-cell activation and proliferation [17]. A potent and specific MCT-1 inhibitor blocks T-cell proliferation and functions as an immunosuppressive agent [18]. Therefore, glycolysis in T cells is regulated by CD147; MCT-1, an important regulator of glycolysis, requires CD147 as an ancillary protein in T cells [17]. CD147 is involved in the development, activation, proliferation, migration, invasion, and adhesion of T cells and it is critical for energy metabolism [19]. In patients with rheumatoid arthritis, CD147 promotes the differentiation of Th17 cells [20].

We speculated that CD147 is also involved in the pathogenesis of Th17-cell-mediated immune disorders and investigated its role in the development of psoriasis. Our study included human samples and samples from CD147-null mice and bone marrow chimeric mice lacking CD147 in their hematopoietic cells of the myeloid lineage.

## 2. Results

### 2.1. Serum Level of CD147 in Patients with Psoriasis

**Figure 1:**
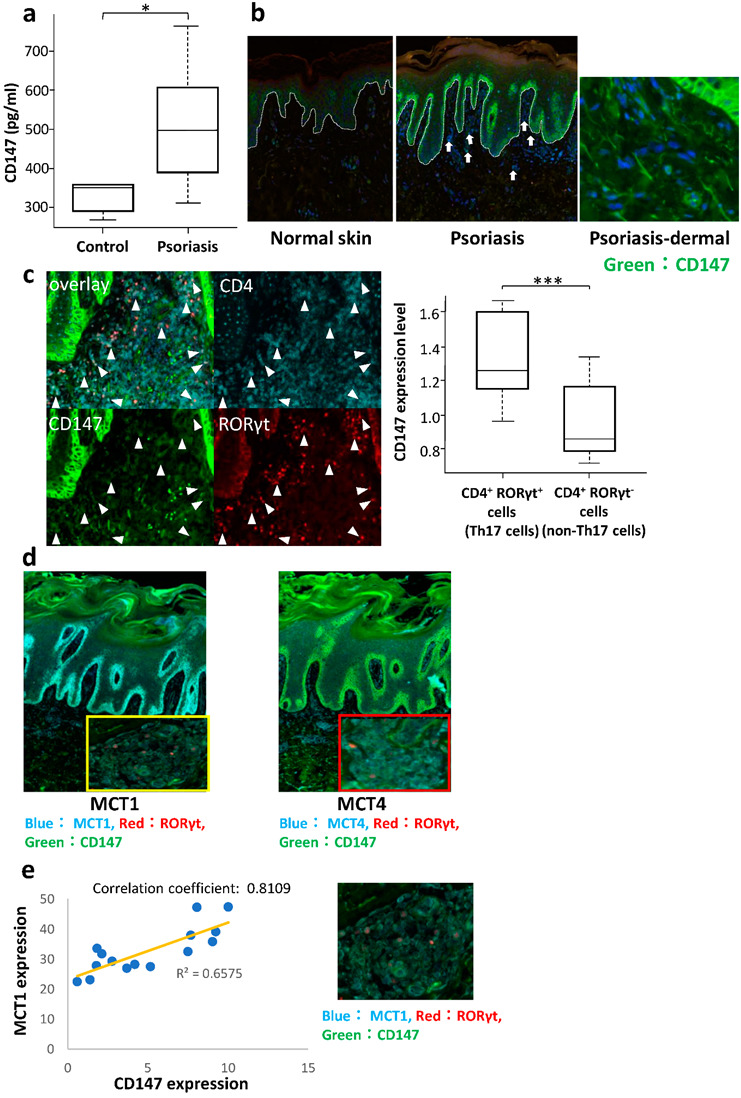
CD147/basigin is elevated in patients with psoriasis. (**a**) Serum CD147 levels in 25 patients with psoriasis and 5 healthy subjects measured using ELISA—The level is significantly higher in patients than in healthy controls. * *p* = 0.0225, Mann–Whitney U test. (**b**) CD147 and MCT expression in dermal psoriasis lymphocytes—CD147 expression is higher in dermal inflammatory infiltrates (arrows) than in normal skin. (**c**) Examination of 14 psoriasis samples showed that CD147 expression is significantly higher in CD4^+^ RORγt^+^ than in CD4^+^ RORγt^−^ T cells. Arrowheads indicate CD147^+^ CD4^+^ RORγt^+^ cells. *** *p* = 0.0009714, Wilcoxon test. (**d**) MCT-1 and MCT-4 are colocalized with CD147 and RORγt^+^ T cells. Hyper-magnified views revealed MCT and CD147 colocalization on RORγt^+^ cells in the dermis. (**e**) MCT-1 expression is positively correlated with CD147 expression in RORγt^+^ T cells in psoriasis patients.

In order to determine whether CD147 is increased in the sera of psoriasis patients, we examined the serum level of CD147 by ELISA. ELISA showed that the mean serum CD147 level was 381.0 ± 119.2 pg/mL (range 274–609 pg/mL) in the 5 healthy subjects and 509.4 ± 126.5 pg/mL (range 377–774 pg/mL) in the 25 psoriasis patients (*p* = 0.0225, Figure 1a). 

### 2.2. Expression of CD147 and MCTs on the Lymphocytes in the Lesional Skin of Psoriasis

To investigate whether CD147 is involved in the development of psoriasis and its association with MCTs, we immunohistochemically examined its expression on lymphocytes in the lesional skin of psoriasis patients. CD147 was highly expressed in the basal cells of the epidermis. Expression of CD147 in the dermal inflammatory infiltrates is higher in psoriasis patients compared with healthy subjects. (Figure 1b). Immunofluorescent staining showed that the expression of CD147 was significantly higher in CD4^+^ RORγt^+^ than in CD4^+^ RORγt^−^ T cells (Figure 1c). MCT-1 and MCT-4 colocalized with CD147 and RORγt^+^ T cells (Figure 1d). A comparison of the expression of CD147 and of MCTs on RORγt^+^ T cells showed that in patients with psoriasis, the numbers of CD147 expressing and MCT-1 expressing RORγt^+^ T cells were correlated. MCT-4 was also expressed on RORγt^+^ T cells, but there was no significant correlation with CD147 expression (Figure 1e).

### 2.3. Effect of CD147 Deficiency on MCT-1 and RORγt expression in CD4^+^ T Cells—In Vitro Studies

**Figure 2:**
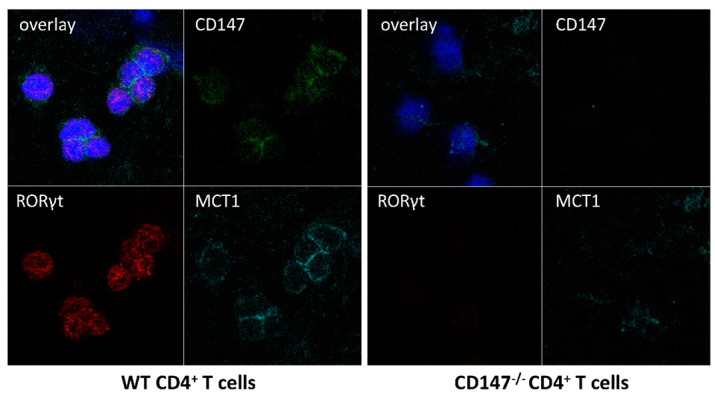
In vitro effect of CD147 deficiency on the expression of MCT-1 and RORγt in CD4+ T cells. Naïve CD4^+^ T cells isolated from the spleens of CD147^−/−^ mice and their WT counterparts were cultured on chamber slides and stimulated with IL-6 and TGF-β. RORγt expression is seen in WT CD4+ T cells, while it is significantly lower in the CD147^−/−^CD4^+^ T cells. MCT-1 is expressed on the plasma membrane of WT CD4^+^ but not on that of CD147^−/−^ CD4^+^ T cells.

Naïve CD4^+^ T cells isolated from spleens of the CD147^−/−^ mice and their WT counterparts were cultured on chamber slides in medium supplemented with antibodies against IFN-γ and IL-4 to prevent their differentiation into Th1 and Th2 cells. The cells were then stimulated with IL-6 and TGF-β to promote their differentiation into Th17 cells. The expression of RORγt, the key transcription factor for the differentiation into Th17 cells, was induced in WT CD4^+^ T cells; its expression was significantly lower in CD147^−/−^ CD4^+^ T cells. MCT-1 was expressed on the plasma membrane of WT CD4^+^ but not on that of CD147^−/−^ CD4^+^ T cells (Figure 2). 

### 2.4. Imiquimod-Induced Psoriatic Lesions in Model Mice

**Figure 3:**
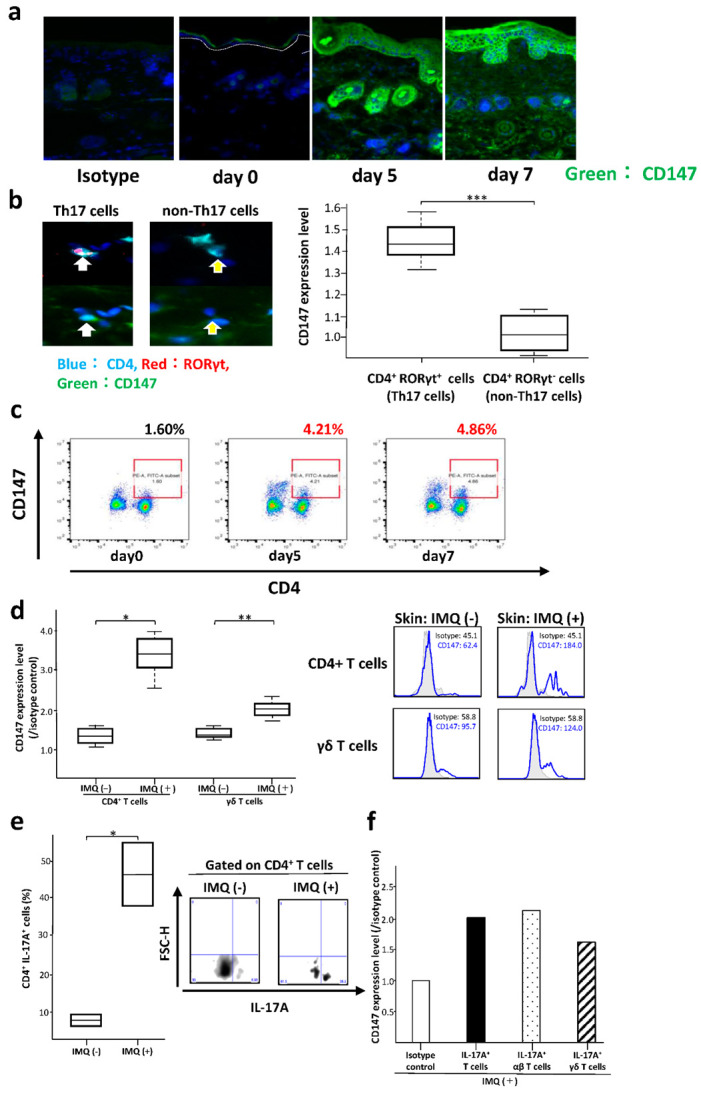
Induction of lesions by imiquimod (IMQ) in a mouse model of psoriasis. (**a**) CD147 expression is upregulated in the IMQ-induced psoriatic lesion. (**b**) CD147 expression is significantly higher in CD4^+^ RORγt^+^ than in CD4^+^ RORγt^−^ T cells. Arrows indicate CD147^+^ CD4^+^ RORγt^+^ T cells (left panels) and CD4^+^ RORγt^−^ T cells (right panels). *** *p* = 0.00085, two-tailed Student’s *t*-test. (**c**) CD147 expression is increased in the splenic CD3^+^ CD4^+^ T cells from mice with IMQ-induced psoriasis. (**d**) CD147 expression is induced by IMQ treatment in CD4^+^ T cells and γδ T cells (*n* = 3). ** *p* = 0.009944, * *p* = 0.04061, two-tailed Student’s *t*-test test. A representative flow cytometry profile is shown. (**e**) The percentage of IL-17A-producing cells is increased by IMQ treatment in skin CD4^+^ T cells from WT mice (*n* = 2). Similar results were obtained in 2 independent mice. * *p* = 0.03557, two-tailed Student’s *t*-test. A representative flow cytometry profile is shown. (**f**) CD147 expression is remarkably induced in IL-17A-producing T cells of all 3 mice examined; the induction is predominant in αβ T cells compared with γδ T cells.

Imiquimod (IMQ)-induced dermatitis is an accepted mouse model of psoriasis. We shaved the whole dorsal skin of C57BL/6L mice and treated with IMQ. The application of 62.5 mg of 5% IMQ cream for seven consecutive days induced psoriatic lesions with hyper-parakeratosis, acanthosis, and dermal infiltration of inflammatory cells (Supplementary Figure S1). CD147 expression was elevated in the lesions (Figure 3a); it was significantly higher in CD4^+^ RORγt^+^ than in the CD4^+^ RORγt^−^ T cells (Figure 3b), and the levels were elevated in the splenic CD3^+^ CD4^+^ T cells (Figure 3c). 

We next prepared the skin single-cell suspensions from WT mice and analyzed CD147 expression in the CD4^+^ T cells and γδ T cells using flowcytometry. CD147 expression was induced by IMQ treatment in CD4^+^ T cells and γδ T cells (Figure 3d). The percentage of IL-17A-producing cells was increased in IMQ-treated CD4^+^ T cells (Figure 3e). CD147 expression was remarkably induced in IL-17A-producing T cells of all 3 mice examined; the induction was predominant in αβ T cells compared with γδ T cells. (Figure 3f).

### 2.5. Effect of CD147 Deficiency on IMQ-Induced Dermatitis

**Figure 4:**
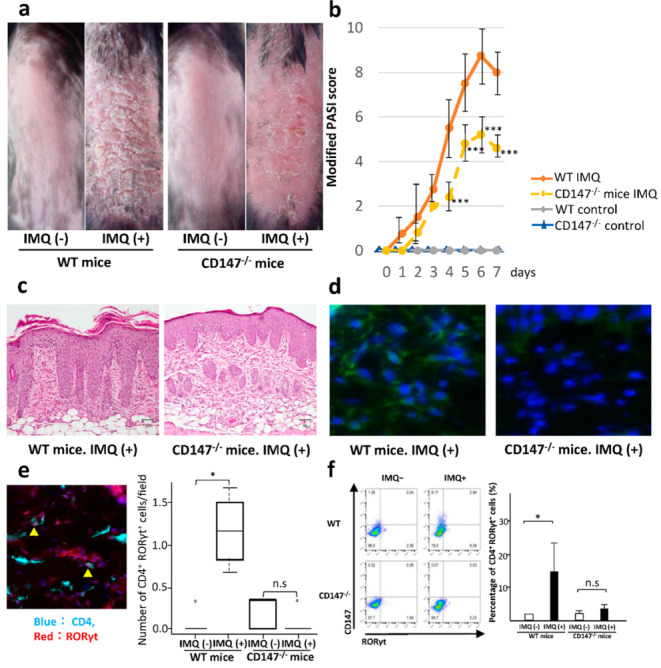

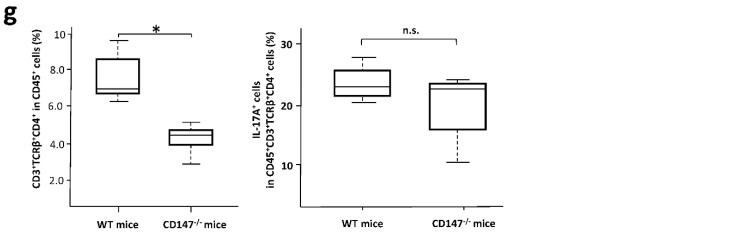
Effect of CD147 deficiency on the development of Imiquimod (IMQ)-induced dermatitis. (**a**) Erythema, induration, and scaling are milder in CD147^−/−^ than in WT mice (day 7). (**b**) Modified PASI scores recorded during the 7-day administration of IMQ. *** *p* < 0.001 (4 WT vs 5 CD147^−/−^ mice), two-way ANOVA test. Significant differences were recorded on days 4, 5, 6, and 7. (**c**) Histological findings in IMQ-treated WT and CD147^−/−^ mice. (**d**) Expression of MCT-1 in IMQ-treated WT and CD147^−/−^ mice. (**e**) The number of CD4^+^ RORγt^+^ T cells in IMQ-treated WT- mice and CD147^−/−^ mice and in IMQ-untreated mice. * *p* = 0.01508, Wilcoxon test. (**f**) Percentage of splenic CD4^+^ RORγt^+^ T cells in IMQ-treated and untreated WT and CD147^−/−^ mice. * *p* = 0.043, Wilcoxon test. (**g**) In skin single-cell suspensions, the percentage of CD3^+^ TcRβ^+^ CD4^+^ T cells in CD45^+^ cells is significantly higher in IMQ-treated WT mice compared with IMQ-treated CD147^−/−^ mice. The frequency of CD45^+^ CD3^+^ TcRβ^+^ CD4^+^ IL-17A^+^ cells is not significantly different between WT and CD147^−/−^ mice (3 WT vs. 3 CD147^−/−^ mice). * *p* = 0.0433, two-tailed Student’s *t*-test. n.s., not significant.

After treating WT and CD147^−/−^ mice for 7 days with IMQ, we noted that on day 7, erythema, induration, and scaling were milder in CD147^−/−^ than in the WT mice (Figure 4a). In CD147^−/−^ mice, the sub-scores were 62.2% for erythema, 88.8% for induration, and 34.2% for scaling, compared to those in WT mice. The modified PASI score was 8.0 ± 0.7 for WT and 4.6 ± 0.5 for CD147^−/−^ mice (*p* < 0.001). Significant intergroup differences were observed on days 4, 5, 6, and 7 (Figure 4b). 

For histological evaluation, skin samples from IMQ-treated mice were harvested on day 7. The skin of WT mice exhibited the characteristic features of psoriasis, including parakeratosis, acanthosis, and dermal infiltration of immune cells. These histological manifestations were milder in CD147^−/−^ mice (Figure 4c). Immunofluorescent staining showed that following the IMQ treatment, the expression of MCT-1 was absent in CD147^−/−^ mice (Figure 4d); the number of CD4^+^ RORγt^+^ T cells increased significantly in WT-, but not in CD147^−/−^ mice treated or not treated with IMQ (Figure 4e).

To investigate whether CD147 promotes the differentiation of CD4^+^ T cells into Th17 cells, we used flow cytometry to compare the expression of RORγt on splenic CD4^+^ cells from WT and CD147^−/−^ mice. In IMQ-treated WT mice, the percentage of splenic CD4^+^ RORγt^+^ T cells was significantly increased. In CD147^−/−^ mice treated or not treated with IMQ, the population of splenic CD4^+^ RORγt^+^ T cells was as low as that in IMQ-untreated WT mice (Figure 4f). In skin single-cell suspensions, the percentage of CD3^+^ TcRβ^+^ CD4^+^ T cells in CD45^+^ cells was significantly higher in IMQ-treated WT mice compared with IMQ-treated CD147^−/−^ mice. The percentage of IL-17A-producing cells in CD45^+^ CD3^+^ TcRβ^+^ CD4^+^ cells was not significantly different between WT and CD147^−/−^ mice, indicating that the number of IL-17A-producing cells is proportional to the percentage of CD3^+^ TcRβ^+^ CD4^+^ T cells, i.e., the number of IL-17A-producing cells is decreased in CD147^−/−^ mice (Figure 4g).

### 2.6. Effect of CD147 Deficiency in Hematopoietic Cells on the Development of IMQ-Induced Dermatitis

To examine the importance of immune cells in psoriasis, we produced bone marrow chimeric mice lacking CD147 in the hematopoietic cells of the myeloid lineage. The protocol for inducing psoriasis in chimeric mice was similar to that used in the murine psoriasis model.

**Figure 5:**
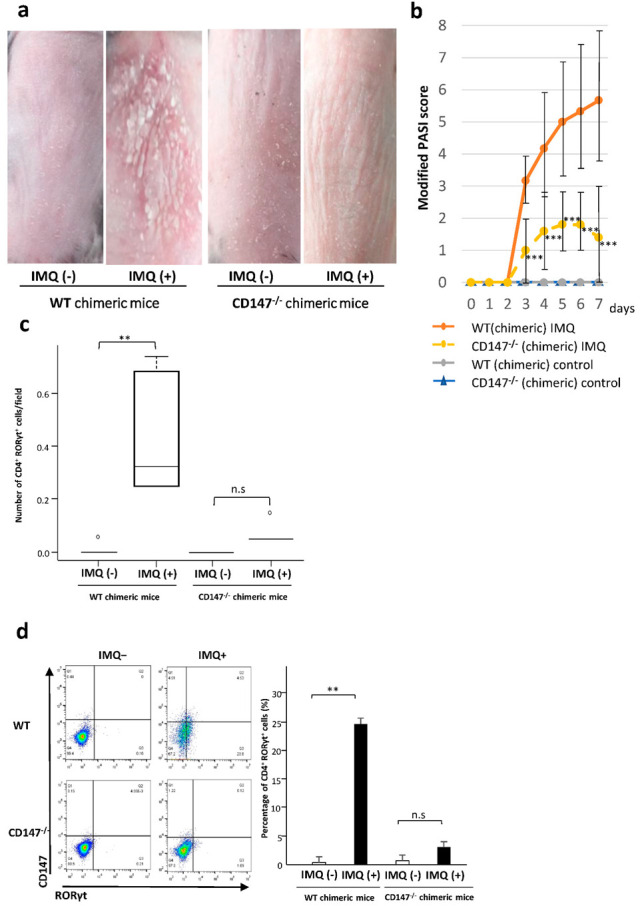
Effect of CD147 deficiency in the hematopoietic cells on Imiquimod (IMQ)-induced dermatitis. (**a**) IMQ-induced dermatitis is milder in CD147^−/−^ chimeric than in WT chimeric mice (day 4). (**b**) Modified PASI scores recorded during the 7-day administration of IMQ. *** *p* < 0.001 (6 WT vs 5 CD147^−/−^ mice), two-way ANOVA test. (**c**) Dermal CD4^+^ RORγt^+^ T cells in WT chimeric- and CD147^−/−^ chimeric mice. ** *p* = 0.003601, Wilcoxon test. n.s., not significant. (**d**) Percentage of splenic CD4^+^ RORγt^+^ T cells in IMQ-treated and untreated WT chimeric- and CD147^−/−^ chimeric mice. ** *p* = 0.004277, Welch test. n.s., not significant.

In the recipients of bone marrow lumps from CD147^−/−^ mice and their WT counterparts, the engrafted donor myeloid cells were 64.8% from WT and 58.1% from CD147^−/−^ donor mice (Supplementary Figure S2). On day 4 after the delivery of donor myeloid cells, IMQ-induced skin lesions appeared milder in the CD147^−/−^ chimeric mice than in the WT chimeric mice (Figure 5a). On day 6, the sub-scores were 43.6% for erythema, 10% for induration, and 53.3% for scaling in CD147^−/−^ chimeric mice, compared to those in the WT chimeric mice. The modified PASI score was 5.33 ± 0.80 in WT chimeric- and 1.80 ± 0.37 in CD147^−/−^ chimeric mice. Significant intergroup differences were observed on days 4–7 (Figure 5b). Following the IMQ treatment, the number of dermal CD4^+^ RORγt^+^ T-cells and the percentage of splenic CD4^+^ RORγt^+^ T cells were significantly higher in WT chimeric-mice than in CD147^−/−^ chimeric mice (Figure 5c,d).

### 2.7. Effect of AP-9 on the Development of IMQ-Induced Dermatitis

**Figure 6:**
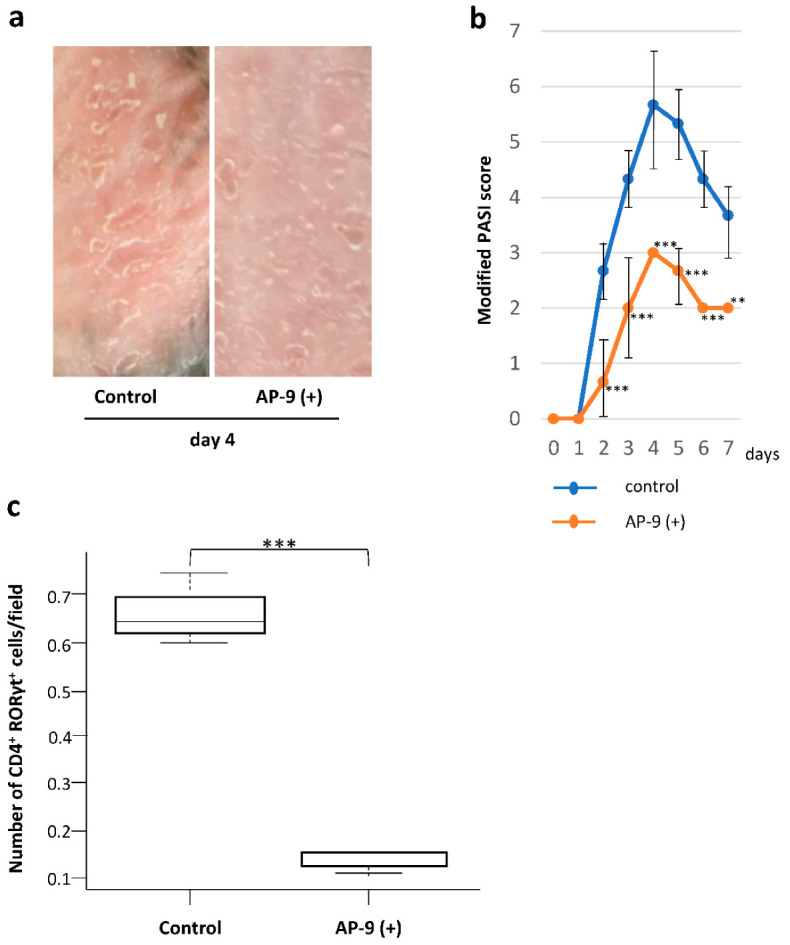
Effect of the CD147 inhibitor (AP-9) on Imiquimod (IMQ)-induced dermatitis. (**a**) IMQ-induced dermatitis was inhibited by AP-9. (**b**) Modified PASI scores recorded during the 7-day administration of IMQ plus AP-9. ** *p* < 0.01, *** *p* = 0.001, two-way ANOVA test. (**c**) Number of dermal CD4^+^ RORγt^+^ T cells induced by IMQ treatment alone and by exposure to AP-9 plus IMQ. *** *p* = 0003479, two-tailed Student’s *t*-test.

We examined the effect of the CD147 antagonist, AP-9, on IMQ-induced dermatitis. AP-9 is a polypeptide which binds specifically to CD147 and significantly inhibits the function of CD147 [21]. When mice were topically treated with AP-9 prior to each application of IMQ for the 7 consecutive days, the development of dermatitis was inhibited (Figure 6a). On day 6, the modified PASI score was 4.33 ± 0.33 in AP-untreated controls and 2.00 ± 0 in AP-9-treated mice (Figure 6b). The number of murine dermal CD4^+^ RORγt^+^ T cells increased by IMQ treatment was significantly decreased by AP-9 treatment (Figure 6c). 

## 3. Discussion

This study demonstrated that CD147is involved, at least in part, in the development of psoriasis. In patients with psoriasis, the serum level of CD147 and the expression of CD147 on their dermal CD3^+^ T cells were increased. In addition, CD147 expression was significantly higher in the CD4^+^ RORγt^+^ Th17-cells than in the CD4^+^ RORγt^−^ T cells. MCT-1 expression in RORγt^+^ T cells was upregulated, and it positively correlated with CD147 expression.

Naïve CD4^+^ T cells from CD147^−/−^ mice showed a low potential for differentiation into Th17 cells, in response to stimulation with IL-6 and TGF-β. These findings indicate that in association with MCT-1, CD147 modulates CD4^+^ T-cell differentiation into Th17 cells. Our study on mice deficient in the CD147 gene yielded direct evidence that CD147 plays a major role in the development of psoriasis. We also demonstrated that IMQ-induced skin inflammation was significantly milder in CD147^−/−^ mice and that AP-9 alleviated IMQ-induced psoriatic symptoms.

CD147 is expressed on dermal lymphocytes and epidermal keratinocytes. Therefore, to confirm the importance of immune cells in the pathogenesis of psoriasis, we produced bone marrow chimeric mice lacking CD147 in the hematopoietic cells of the myeloid lineage. Chimeric mice showed poor response to IMQ. The reduction in the modified PASI scores of CD147^−/−^ chimeric- and CD147^−/−^ mice was comparable. These results show that CD147 in immune cells is responsible for the development of psoriasis as well as CD147 expressed in the epidermal keratinocytes.

CD147 is a ubiquitously distributed cell surface glycoprotein with a single transmembrane domain, two extracellular immunoglobulin-like domains, and a short C-terminal cytoplasmic tail [7]. MCT-1 directly binds to the transmembrane and cytoplasmic regions of CD147 [10,22]. CD147 colocalizes with MCT-1, and a CD147-targeting siRNA clearly abrogated the expression of MCT-1 and CD147 in human malignant melanoma cells. These cells expressed higher levels of CD147, and their glycolysis rate was higher than that of normal melanocytes. The siRNA-mediated silencing of CD147 downregulated glycolysis, indicating that in malignant melanoma (MM) cells, CD147 modulates glycolysis by regulating lactic acid transport in association with MCT-1 [12]. Our findings suggest that the same mechanism underlies the pathogenesis of psoriasis. CD147 interacts with MCT-1 to promote T-cell glycolysis, which is essential for Th17 cell differentiation, a pivotal immunopathological feature in psoriasis.

We also showed that the serum level of CD147 was increased in patients with psoriasis, suggesting the existence of a soluble form of CD147. Soluble CD147 in the serum or plasma was found in patients with psoriasis [23], systemic lupus erythematosus (SLE) [24], and MM [25]. In patients with psoriasis, the serum level of soluble CD147 was significantly higher than that in the healthy controls. The levels varied with the psoriasis type; it was low in plaque-type psoriasis and was higher in patients with pustular psoriasis, psoriatic arthritis, and psoriatic erythroderma [23]. Plasma CD147 levels in patients with SLE accurately reflect the histological activity of lupus nephritis. In inflammatory immune diseases, soluble CD147 are probably derived from lymphocytes and leukocytes of the myeloid lineage, including neutrophils and macrophages. In psoriasis, CD147 is highly expressed on peripheral blood neutrophils and its expression level is significantly correlated with the PASI score [26]. Taken together, these observations suggest that the serum level of CD147 may be a biomarker for various diseases, including psoriasis.

In conclusion, using CD147^−/−^ mice and bone marrow chimeric mice lacking CD147 in their hematopoietic cells, we demonstrated for the first time based on the findings obtained in this study, we speculate that CD147 contributes to the development of psoriasis via the regulation of T cell glycolysis in association with MCT-1 and that it promotes Th17 cell differentiation. Ap-9 treatment inhibited the development of dermatitis in mice exposed to IMQ, suggesting that CD147 is a promising therapeutic target in patients with psoriasis.

## 4. Materials and Methods

### 4.1. Ethical Considerations

All human study protocols were approved by the Human Investigation Committee of the Kagoshima University Graduate School of Medical and Dental Sciences. We adhered strictly to the Declaration of Helsinki throughout this study. All animal experiments were approved by the Ethics Committee for Animal Experimentation at Kagoshima University. 

Sera and skin specimens were obtained with prior written informed consent from 5 healthy subjects and 25 psoriasis patients with a histopathological diagnosis of psoriasis.

### 4.2. Enzyme-Linked Immunosorbent Assay (ELISA)

Peripheral blood was obtained from human subjects, and the serum level of CD147 was determined using the human EMMPRIN/CD147 immunoassay kit (R&D Systems, Minneapolis, MN, USA), according to the manufacturer’s instructions. The absorbance at 450 nm was measured using an ELISA reader (Multiskan FC Microplate Photometer, Thermo Fisher Scientific, Tokyo, Japan).

### 4.3. Animals

*Bsg* is a symbol for the CD147/basigin gene. As Bsg^-/-^ mice are rarely born after ordinary mating, mice deficient in the gene were produced as described previously [27,28]. Briefly, Bsg^+/−^ mice on the 129/SV background were backcrossed with C57BL/6J mice to generate F1 hybrid offspring (reverse F1 hybrid). Intercrossing these mice resulted in mixed reverse F2 mice (*Bsg-null* mice); they are hereafter referred to as CD147^−/−^ mice. The mice used in this study were 8–10 weeks old and weighed 20–25 g. They were housed in a controlled environment, and they received standard food and water.

To produce bone marrow chimeric mice lacking CD147 in the hematopoietic cells, Ly5.1 recipient mice were exposed to 10 Gy of total body irradiation. Bone marrow lumps were obtained from the femur of CD147^−/−^ mice and their wild type (WT) counterparts, and the lumps of bone marrow were transplanted to the recipient mice transvenously. Leukocytes from the Ly5.1 recipients and the C57BL/6J donors expressed CD45.1 and CD45.2, respectively.

### 4.4. Induction of Psoriasis in Mice

After shaving the dorsal skin of CD147^−/−^ mice (*n* = 5), 62.5 mg of 5% imiquimod (IMQ) cream (Mochida Pharmaceutical Co., Tokyo, Japan) was applied for 7 consecutive days. The controls, WT mice (*n* = 5), were shaved but not treated with IMQ. During the experiment, one mouse treated with IMQ died. Clinical skin scores were recorded daily. Skin, serum, and spleen samples were obtained on Day 7. The degree of skin inflammation was evaluated using the cumulative disease severity scoring system modified from the psoriasis area and severity index (PASI) score. The degree of skin erythema, induration, and scaling was classified as 0 = none, 1 = mild, 2 = moderate, 3 = severe, and 4 = maximum. These scores were summed, resulting in a theoretical maximum score of 12.

### 4.5. Light and Confocal Immunofluorescence Microscopy

Human samples were formalin-fixed, paraffin-embedded, and stained with mouse IgG1 anti-CD147 antibody (Ab) (1:100, BioLegend, San Diego, CA, USA, catalog 306202), mouse IgG2a anti-RORγt Ab (1:500, Sigma Aldrich, Tokyo, Japan catalog MABF81), rabbit anti-CD4 Ab (1:50, Abcam, Cambridge, UK, catalog 213215), mouse IgG2b anti-MCT-1 Ab (1:100, Sigma Aldrich, catalog 2702323), and mouse IgG2b anti-MCT-4 Ab (1:50, Santa Cruz, Dallas, TX, USA, catalog 376139). The secondary antibodies for CD147, CD4, and MCTs were Af488-conjugated donkey anti-mouse IgG1 Ab (1:200, Jackson ImmunoResearch, Baltimore, PA, USA, catalog 715-546-151), Cy5-conjugated goat anti-mouse IgG2b Ab (1:200, Jackson ImmunoResearch, catalog 115-175-207), and Cy5-conjugated donkey anti-rabbit IgG Ab (1:200, Jackson ImmunoResearch, catalog 711-175-152). RORγt was visualized using the TSA tetramethylrhodamine system (PerkinElmer, Boston, MA, USA, catalog NEL702001KT).

Mouse samples were stained with Af488-conjugated rat IgG1 anti-CD147 Ab (1:20, BioLegend, catalog 123718), APC-conjugated rat IgG2b anti-CD4 Ab (1:20, BioLegend, catalog 100411), rabbit IgG anti-RORγt Ab (1:60, Abcam, catalog 207082), and guinea pig IgG anti-MCT-1 Ab (1:50, Frontier-Institute, Tokyo, Japan, catalog GP-Af950). The secondary antibodies for RORγt and MCT-1 were TRITC-conjugated donkey anti-rabbit IgG Ab (1:200, Jackson ImmunoResearch, catalog 711-025-152), and Cy5-conjugated donkey anti-guinea pig IgG Ab (1:200, Jackson ImmunoResearch, catalog 706-175-148).

Nuclei were stained with 4′,6-diamidino-2-phenylindole (DAPI; 1:500, Dojindo, Tokyo, Japan, catalog 340-07971). Fluorescence images were acquired using a laser-scanning microscope (BZ-X700; Keyence, Osaka, Japan) and analyzed using the ImageJ software (National Institutes of Health, Bethesda, MD, USA). The observation range was 10 fields at 400× magnification.

Naïve CD4^+^ T cells (1 × 10^6^) in culture medium, IMDM supplemented with 10% fetal bovine serum (FBS), 50µM 2-mercaptoethanol, and 5 ng/mL Antibiotic-Antimycotic solution, (1 mL/well) were cultured on 24-well Lab-Tek Chamber Slides (Thermo Fisher, Tokyo, Japan), treated with plate-bound anti-CD3 Ab (2 μg/mL; BioLegend catalog 100340) and anti-CD28 Ab (2 μg/mL; BioLegend, catalog 102116) for 3 h at 37 °C. They were stimulated with TGF-β (2 ng/mL, R&D, catalog MAB1835R-SP) and IL-6 (20 ng/mL, R&D, catalog 406-ML-005) in the presence of anti-IFN-γ Ab (10 μg/mL; BioLegend, catalog 505834) and anti-IL-4 Ab (10 μg/mL; BioLegend, catalog 504122), acetone-fixed (10 min), air-dried (30 min), and washed twice in PBST (Tween 20). Non-specific binding was blocked by incubating with 10% donkey serum in PBST for 30 min. The primary antibodies used were rabbit anti-RORγt Ab (1:60, Abcam, catalog 207082), Af488-conjugated anti- CD147 Ab (1:20, BioLegend, catalog 123718), and guinea pig anti-MCT-1 Ab (1:50, Frontier Institute, catalog GP-Af950). The secondary antibodies were TRITC-conjugated donkey anti-rabbit IgG Ab (1:200, Jackson ImmunoResearch, catalog 711-025-152) and Cy5-conjugated donkey anti-guinea pig IgG Ab (1:200, Jackson ImmunoResearch, catalog 706-175-148). Images were acquired with a confocal laser scanning microscope (Zen LSM 70; Carl Zeiss, Oberkochen, Germany) using a 63 × oil objective.

### 4.6. Preparation of Skin Single-Cell Suspensions

Full-thickness dorsal skin was excised, washed in PBS, and then incubated in RPMI containing 0.4 mg/mL of Liberase TL (Roche, Penzberg, Germany, catalog 5401020001) at 37 °C for 1.5 h. For the final 15 min., 0.05% DNase I (final concentration) was added. Single cell suspensions were prepared by vigorous trituration of skin digests with 60 mL syringes, followed by sequential filtration through 100, 70, and 40 μm nylon mesh. Red blood cells and dead cells were eliminated using Lympholyte-M (Cedarlane, Ontario, Canada, catalog CL5035) according to the manufacturer’s instructions.

### 4.7. Flow Cytometry

Lymphocytes isolated from mouse spleens were fixed with Cytofix (BD Biosciences, Tokyo, Japan) and stained with the following antibodies for 30 min at 4 °C in the dark: FITC-conjugated anti-mouse CD147 Ab (BioLegend, catalog 123705), PC5.5-conjugated anti-mouse CD3 Ab (Invitrogen, Thermo Fisher Scientific, Tokyo, Japan, catalog 35-0031-82), APC-Cy7-conjugated anti-mouse CD4 Ab (BioLegend, catalog 100528), and APC-conjugated anti-mouse RORγt Ab (Invitrogen, catalog 17-6983-82). Lymphocytes isolated from the peripheral blood of chimeric mice were fixed with Cytofix (BD Biosciences) and stained with FITC-conjugated anti-mouse CD45.1 Ab (BioLegend, catalog 110705) and APC-conjugated anti-mouse CD45.2 Ab (BioLegend, catalog 109813) for 30 min at 4 °C in the dark. Viable cells were analyzed on an EPICS flow cytometer (Beckman Coulter, Fullerton, CA, USA), and cell populations were determined using FlowJo software (Tree Star, San Carlos, CA, USA).

For the analysis of the skin single-cell suspensions, the following monoclonal antibodies were used; anti-mouse CD147 (BioLegend, catalog 123705), anti-mouse CD45 (BioLegend, catalog 103111), anti-mouse CD4 (BioLegend, catalog 100407), anti-mouse TCRβ (BioLegend, catalog 109223), and anti-mouse TCRγδ (BioLegend, catalog 118119) and anti-mouse CD3e (Invitrogen, catalog 35-0031-82) and anti-mouse IL-17A (Invitrogen, catalog 25-7177-80). CD4^+^ T cells and γδ T cells were identified by gating CD45^+^CD3^+^TCRβ^+^CD4^+^ cells and CD45^+^CD3^+^TCRγδ^+^ cells, respectively.

### 4.8. Intracellular IL-17A Staining

Skin single-cell suspension was stimulated with eBioscience™ Cell Stimulation Cocktail (plus protein transport inhibitors) (500×) (Invitrogen, Carlsbad, CA, USA) at 37°C for 5 h. Following surface antigen staining, fixation and permeabilization were carried out with BD Cytofix/Cytoperm™ (BD Biosciences, San Jose, CA, USA) according to the manufacturer’s instructions.

### 4.9. Treatment with CD147 Inhibitor

The CD147 antagonist peptide-9 (AP-9), purchased from Sangon Biotech (Shanghai, China), was used to inhibit the action of CD147. The amino acid sequence of AP-9 was Tyr-Lys-Leu-Pro-Gly-His-His-His-His-Tyr-Arg-Pro [21]. AP-9 (10 mg) was mixed with 600 mg of petrolatum-based ointment containing 5% stearyl alcohol. We topically applied 10 mg of the ointment along with the IMQ treatment, for inhibiting IMQ, for seven consecutive days.

### 4.10. Statistical Analysis

R software (R Development Core Team, Auckland, NZ, USA) was used for statistical analyses. Values are presented as the mean ± SE. We compared the mean values between two groups using the two-tailed unpaired Student’s *t*-test for data showing normal distribution and the two-tailed Wilcoxon test for data not showing normal distribution or revealing unequal variances. Statistical significance was set at *p* < 0.05.

## Data Availability

Not appliable.

## Supplementary Materials

The following are available online at https://www.mdpi.com/article/10.3390/ijms23010177/s1.

## Abbreviations

AP-9: CD147 antagonist peptide; ATP, Adenosine triphosphate; Ab, Antibody; ELISA, En-zyme-linked immunosorbent assay; IMQ, Imiquimod; IL, Interleukin; MM, Malignant melanoma; MCT, Monocarboxylate transporter; PASI, psoriasis area and severity index; RORγt, Retinoic acid receptor-related orphan nuclear receptor γt; SLE, Systemic lupus erythematosus; TGF-β, Trans-forming growth factor-β; WT, Wild-type.
