# Supplementary material for: CD147 Is Essential for the Development of Psoriasis via the Induction of Th17 Cell Differentiation
Source: Int J Mol Sci. 2021 Dec 24;23(1):177. doi: 10.3390/ijms23010177 (PMC8745261; doi:10.3390/ijms23010177)
Supplement: Supplementary file 1 [file ijms-23-00177-s001.zip › ijms-1506454-supplementary.pdf]

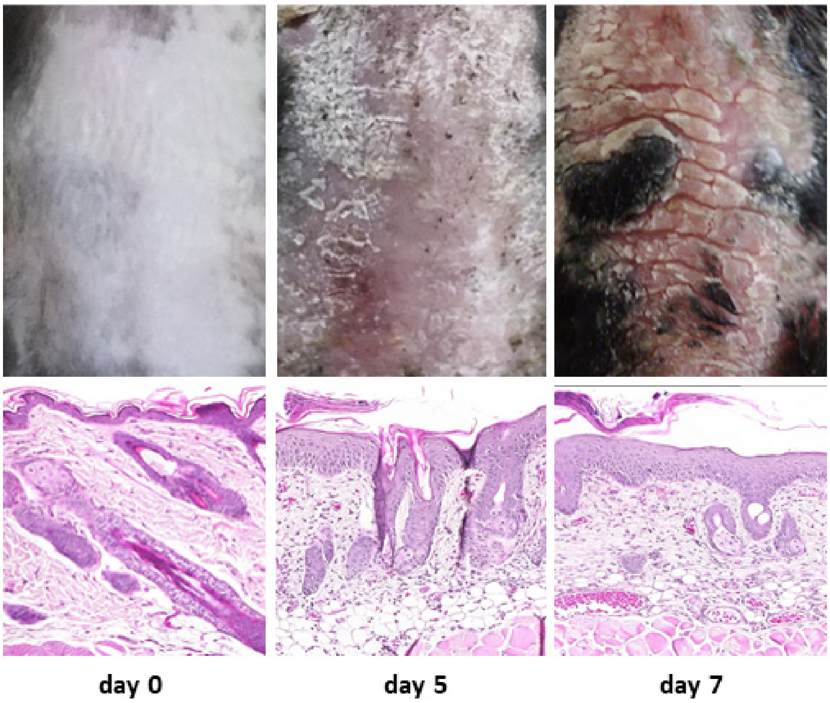

Figure S1. Induction of psoriatic lesions by imiquimod in psoriasis model mice. The 7-day application of 62.5 mg of 5% IMQ cream to the shaved back of mice induced psoriatic lesions with hyper-parakeratosis, acanthosis, and dermal infiltration of inflammatory cells.

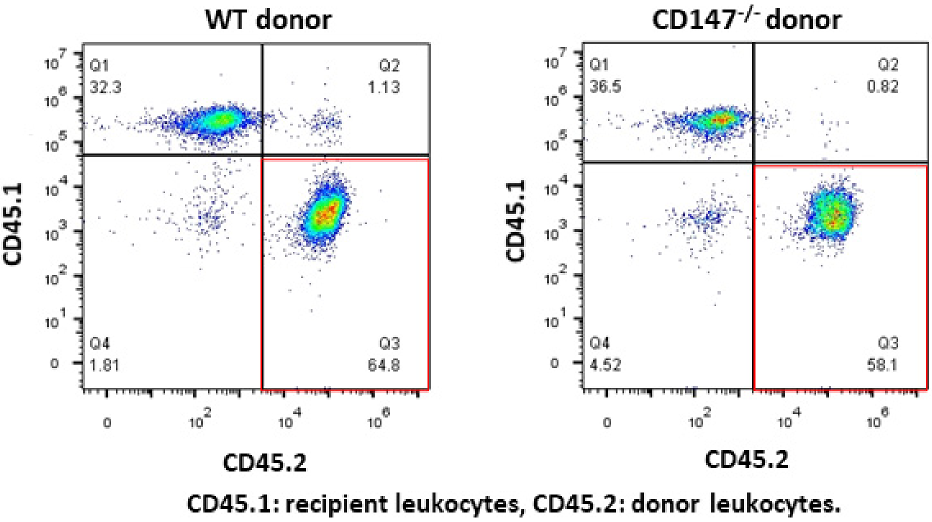

Figure S2. The donor bone marrow chimeric mice lacked CD147 hematopoietic cells of myeloid lineage. In the recipient mice, successfully engrafted donor myeloid cells were 64.8% from WT donor mice and 58.1% from CD147$^{-/-}$ mice.
